# Teaching Medical Spanish to Improve Population Health: Evidence for Incorporating Language Education and Assessment in U.S. Medical Schools

**DOI:** 10.1089/heq.2019.0028

**Published:** 2019-11-01

**Authors:** Pilar Ortega, Norma Pérez, Brenda Robles, Yumirle Turmelle, David Acosta

**Affiliations:** ^1^ Department of Emergency Medicine, College of Medicine, University of Illinois at Chicago, Chicago, Illinois.; ^2^ Department of Medical Education, College of Medicine, University of Illinois at Chicago, Chicago, Illinois.; ^3^ Hispanic Center of Excellence, School of Medicine Special Programs, University of Texas Medical Branch, Galveston, Texas.; ^4^ Language Interpreters Program, National Institutes of Health Clinical Center, Bethesda, Maryland.; ^5^ Department of Pediatrics, Washington University St Louis School of Medicine, St Louis, Missouri.; ^6^ Association of American Medical Colleges, Washington, District of Columbia.

**Keywords:** language concordance, medical Spanish, limited English proficiency, patient/physician communication, Hispanic/Latino health, population health

## Abstract

**Introduction:** Language concordance between patients and physicians is an important factor in providing safe and effective health care, with Spanish as the predominant and fastest growing non-English language in the United Sates. However, despite increasing demand for medical Spanish education, valid concerns about inadvertently increasing provider use of limited Spanish with patients, lack of knowledge of best practice in education and assessment, and lack of institutional support still present barriers to medical Spanish education in medical schools.

**Methods:** The authors conducted a narrative review of existing literature that evaluates the link between medical Spanish education of physicians and language concordance.

**Results:** Medical Spanish educational efforts, although increasing, are not consistently linked to learner assessment. The literature to date supports that for medical Spanish education to improve patient outcomes, it should be linked to assessment methodology that demonstrates improvement in language concordance with Spanish-speaking patients, and should include safety measures to prevent inadvertent communication errors. The authors review data for published medical Spanish postcourse language assessment strategies and provide recommendations to ensure responsible and competent use of medical Spanish skills.

**Conclusion:** The authors propose three structural elements that should be considered when incorporating or enhancing medical Spanish education in medical schools: institutional endorsement of the role of medical Spanish education within a national health disparities context; precourse proficiency testing to establish student starting level; and learner postcourse communications skills and limitations assessment to provide individualized recommendations and assure patient safety.

## Introduction

The question of whether and how to initiate medical Spanish courses in medical school programs is an increasingly common medical education scenario with few evidence-based answers. Students asking the question may be eager to use any pre-existing Spanish skills with patients with the goal to help the underserved.^1^ Medical educators asking the question may be plagued by the uncertainty of their own skills in teaching or assessing medical non-English language skills.^2^ Medical school administrators may wonder how to justify including Spanish language skills in a time-scarce curriculum. All stakeholders are further faced with the question of how to offer medical Spanish education in a way that appropriately considers the quality and patient safety concerns that can arise with partial language knowledge acquisition.^3^ Although medical Spanish courses are increasingly requested in U.S. medical schools,^4^ data are sparse regarding methods of implementation, learner assessment, and confirmation of course effectiveness and sustainability. Therefore, an updated review of existing evidence may facilitate high-quality medical Spanish program implementation and help identify next steps in related educational research.

Language concordance between patients and physicians is an important factor in providing safe and effective health care. Language has been identified as a major contributor to health disparities for patients who prefer languages besides English, and may result in increased risk of medical errors and poor health outcomes.^5^ Prior research and policy efforts to address the need for Spanish language health services have primarily focused on language assistance by means of interpretation services, as federally mandated^6^ and guided through the national standards for culturally and linguistically appropriate services.^7^ However, it has been suggested that overcoming this challenge will also require augmenting the Spanish language competency of physicians themselves through the recruitment of Spanish-speaking physicians and increased training of the physician workforce in conversational and medical Spanish.^3,8,9^ This latter approach has been supported by evidence that demonstrates that physician/patient language concordance is associated with improved quality in health care delivery and health outcomes.^10^ Furthermore, preparing physicians to appropriately communicate with and care for vulnerable linguistic minority patients fits within the existing communication skills and cultural competence standards of the Liaison Committee on Medical Education.^11,12^

Providing medical Spanish educational opportunities for physicians should be approached thoughtfully and should incorporate validated tools for careful assessment of provider skill set. Medical Spanish education should improve quality of care and patient safety without overburdening bilingual physicians or increasing the risk that medical Spanish learners will overestimate their competence in using Spanish skills in patient care without recognizing limitations—a concept known as false fluency.^3,13^ Existing guidelines for medical school medical Spanish courses were established in the medical literature by Reuland et al. in 2008^14^ and other guidelines for medical Spanish courses (including undergraduate and graduate programs) were presented in the language literature by Hardin in 2015.^2^ Recent research shows that there are still significant barriers to establishing courses that meet these criteria,^4^ and that demonstrating course effectiveness and learner competency achievement remains a significant concern. In particular, stakeholders in medical Spanish education, such as medical students and faculty who wish to enhance medical Spanish opportunities at their institutions, may be unequipped with the data and/or strategies to gain institutional support for the initiation of new medical Spanish courses or to improve existing ones.

A recent systematic review highlights the patient outcome benefits of language concordance.^15^ It follows that efforts to improve physician language abilities in a target language, if effective, may also enhance language concordance with the given linguistic minority population and may therefore improve patient outcomes for that population. The purpose of conducting this narrative review was (1) to review existing literature that evaluates the link between medical Spanish education of physicians and language concordance or patient outcomes (i.e., the effectiveness of medical Spanish courses) and (2) to identify existing gaps in knowledge regarding medical Spanish education and assessment.

## Methods

To describe existing knowledge or gaps in knowledge of medical Spanish course effectiveness in the context of population health, we conducted a review of literature. We performed the search through Medline, PubMed, and Google Scholar using the following keywords: medical education, medical Spanish, medical Spanish education, medical Spanish assessment, clinical Spanish, language in health, language concordance, limited English proficiency, public health, population health, and patient outcomes. The review was initially conducted as background information for the Association of American Medical Colleges (AAMC)—Centers for Disease Control Diversity 360 Webinar titled “Teaching Medical Spanish to Improve Population Health,”^16^ and was later expanded and reformatted into this article. Opinions expressed in the review are also based on the authors' professional experience as bilingual physicians, medical interpreters, and medical Spanish educators.

## Results

### Medical Spanish education within a national health disparities context

According to the 2016 U.S. census, the Hispanic/Latino^*^ population is the nation's largest and fastest growing ethnic or racial minority, constituting 17.8% of the total population, and Spanish represents the most widely spoken non-English language.^17^ Of the U.S. population determined by census criteria to have limited English proficiency, 64% are Spanish speaking, and this percentage is expected to rise. It is estimated that the U.S. Latino population will increase by 115% over the next 50 years, and by 2060 will compromise 119 million people, or 29% of the U.S. population.^17^

Even though Latinos make up the largest minority population in the United Sates, they only represent a small fraction (5.2%) of the physician workforce,^18^ and recent national data of medical school graduation rates show that these numbers are not improving, with Latino students representing only 4.6% of U.S. medical school graduates in 2015.^19^ Furthermore, although Latino physician candidates are considerably more likely to report Spanish skills compared with non-Latinos,^20^ they may have never been exposed to medical vocabulary or complex communication scenarios such as procedural consent, delivering bad news, or psychiatric interviewing skills and may be unprepared to provide equivalent quality of care in Spanish as they would in English without specialized training (i.e., medical Spanish).^21^ Even without skills verification, Latino medical students with some Spanish skills report being called upon to interpret or care for patients in Spanish.^1^

Despite federal mandates for provision of health care services in a patient's language by means of qualified professionals, the use of family members or untrained staff, including medical students, as *ad hoc* interpreters remains in widespread unsupervised use and presents serious patient safety risks.^1,22,23^ Most studies regarding linguistically appropriate access to care have focused on interpreter use rather than provider language skills. Although training in use of interpreters remains a critical communication skill for physicians,^24,25^ language concordant care is known to be superior with regard to patient satisfaction and quality measures.^26,27^

Language discordance has been associated with multiple health disparities, including decreased patient satisfaction,^28^ less access to preventive health services,^29,30^ increased risk of medical errors and misdiagnoses,^31^ longer hospital stays, and increased care cost.^5,31,32^ A recent systematic review of 33 available studies that compared language concordant medical care with language discordant medical care with the intervention of trained or *ad hoc* interpreters shows that in the majority of cases, language concordance improves outcomes.^15^

Recent data from the state of California demonstrate that the deficit in the number of language concordant primary care physicians relative to the number of minority language speakers in the population is most significant for the Spanish-speaking population compared with any other linguistic minority group.^33,34^ Equivalent analyses have not been completed for the rest of the United Sates but are expected to demonstrate similar results, given the Spanish-speaking population growth nationwide. Moreover, since physician language proficiency information, when available, mostly consists of self-assessed binary data (e.g., physicians may be asked to report what languages they speak but are not asked to describe proficiency level or take an assessment), the accuracy of existing data may be limited and may underestimate the deficit in the number of physicians competent in independently caring for linguistic minority patients.^8^

In addition to the lack and poor quality of physician language data, patients with non-English language preferences are often excluded from research due to difficulties with informed consent.^35,36^ The current systemic lack of accountability for physician non-English language skills is coupled with the increasingly common reality of the urgent communication needs in patient settings, with the outcome that medical students and physicians may already rely on limited Spanish skills to convey or obtain critical information during patient care.^1,37^ The literature to date supports that for medical Spanish education to improve patient outcomes, it should be linked to assessment methodology that demonstrates improvement in language concordance with Spanish-speaking patients, and should include safety measures to prevent inadvertent communication errors resulting from false fluency.

### Current state of medical Spanish education in U.S. medical schools

The most comprehensive overview regarding existing U.S. medical school medical Spanish courses to date comes from a study by Morales et al. that published the results of their national survey of U.S. medical school deans regarding the availability, characteristics of, and obstacles to establishing medical school medical Spanish courses.^4^ This e-mail-based survey included 39 questions developed based on Reuland's principles.^14^

Over a period of 2 years from 2012 to 2014, 83% (110/130) of institutions responded, with the number of participating medical schools well-represented across the four AAMC-designated geographic regions (northeastern, central, southern, and western). Seventy-three medical schools reported having a medical Spanish curriculum (66%), representing an increase from 48% identified by a prior study in 2005.^38^ Of note, of the 37 schools that reported not having medical Spanish courses, 27% reported having had one previously. The most common barriers cited by medical schools as reasons for not having or discontinuing medical Spanish courses included lack of time in the overall curriculum (51%), cost (28%), overly heterogeneous Spanish-speaking skill level (25%), and insufficient faculty support (20%). Teaching modalities used in the courses were variable (e.g., didactic, role play, standardized patients [SPs], immersion experiences, or online modules), as well as the qualifications of the instructors (e.g., medical faculty students, interpreters, or language instructors).^4^ The finding that medical Spanish courses vary considerably regarding teaching modalities and instructor qualifications is consistent with a review by Hardin and Hardin in 2013, in which the authors reviewed 23 published studies of medical Spanish curricula.^39^

Importantly, most schools that offer medical Spanish education report not having a method to determine students' proficiency before enrolling in the course, nor an assessment method at course completion,^4^ even though both elements have been recommended by experts as critical to best practice in physician language education.^14,39^ Furthermore, 75% of medical schools in the most recent national survey reported allowing their students to perform patient interviews in Spanish, and 57% stated that they did not require any proof of language proficiency.^4^ The lack of assessment of medical Spanish skills before their use in patient care is a major concern that threatens to exacerbate communication errors endangering patient safety.^5,9,20,21,23,40^ Some literature reports use of a commercially available phone-based oral examination to certify physician non-English language abilities before patient care,^41^ but this assessment has not been evaluated for alignment with medical Spanish curricular objectives or learner target competencies, and so, its relevance in examining postcourse outcomes is unknown. Few studies have evaluated physician language assessment in relation to medical Spanish educational interventions.

The lack of knowledge of how to properly assess language proficiency of physicians is a significant reason that medical Spanish educators may not formally assess proficiency. Clinician-educators—the most common teachers of medical Spanish in medical schools^4^—may benefit from combining their clinical expertise in communicating with Spanish-speaking patients with the experience of language educators in addressing learner proficiency assessment.^2,42^ Some medical Spanish experts have published their strategies regarding postcourse proficiency assessment of medical Spanish learners. Of the six publications describing outcomes of medical Spanish courses in medical school or similar settings since 2012, four describe learner assessment via SP objective structured clinical examinations,^43–46^ two of which are medical Spanish courses in medical school programs^43,44^ and two in other health professions graduate programs (pharmacy and physician assistant programs).^45,46^ Two other studies describe use of an oral proficiency interview as a learner assessment tool.^47,48^ A seventh publication that describes medical Spanish curricula and assessment at three medical schools reports use of SP examinations in all three programs and an oral proficiency interview in one of the programs.^11^

Since many medical Spanish learning experiences in medical school are peer-led extracurricular activities rather than formal courses,^4^ it is possible that a significant portion of the existing educational efforts are not being reported, supervised, evaluated for effectiveness, or acknowledged either by individual institutions or interinstitutional curricular surveys.^8^ For example, AAMC's Curriculum Inventory^49^ captured few medical Spanish courses compared with the survey by Morales et al.^4^ since it did not directly inquire about elective courses.

While most studies report improvement in medical Spanish skills following educational interventions,^11,43–48^ not all programs routinely evaluate unintended outcomes—such as false fluency—or train students strategies to recognize and address language discordance if present—such as interpreter use. Such communication skills have been referred to as global linguistic competence due to their relevance in communicating with patients of any language preference, whether a patient/physician language discordance is present or not.^11^ Some medical Spanish courses in graduate medical education programs have reported that some global linguistic learner skill usage may be worsened after courses, specifically noting reduced use of interpreters by learners despite still demonstrating skill limitations, including medical communication errors in Spanish.^50,51^ These findings should alert educators to the potential risks of partial language knowledge acquisition. Of note, these concerning outcomes were both identified following short-duration courses taking place in residency programs. Other residency programs with large Spanish-speaking patient populations have successfully implemented longitudinal medical Spanish educational interventions that integrate intensive medical Spanish classroom learning and daily Spanish use in clinical settings with interpreter support and progressive resident independence.^52^ Furthermore, due to the resident physician's clinical responsibilities, resident physicians may perceive increased urgency to use skills with patients compared with medical students, although additional study is needed to evaluate the potential differences between language education in undergraduate and graduate medical education settings and among intensive, longitudinal, and mixed-structure courses.

## Discussion

Unless the number of competent Spanish-speaking physicians increases over time and all providers are educated on global linguistic skills, the language discordance gap is likely to lead to more health disparities for the U.S. Latino patient population and other linguistic minorities. A vast majority of hospitals nationwide are encountering patients with language preferences besides English on a regular basis,^22^ suggesting that even medical schools and hospitals in cities without large urban populations need to address language discordance. In addition, a medical school's goal should not be solely to prepare their students for the practice of medicine in the particular hospital where they train, but rather to serve the increasingly global population of patients in the United Sates. One potential solution would be to recruit more bilingual students into medical school^3^; however, the number of Latino candidates applying to and graduating from medical schools has remained constant over the last 20 years.^53^ Furthermore, even physician heritage Spanish speakers—individuals who were exposed to or learned Spanish at home but whose proficiency level may significantly vary—may lack the necessary language skills to practice medicine in Spanish.^54^ Therefore, medical schools should focus on teaching medical Spanish to selected candidates and global linguistic competence to all students as part of the medical school curriculum to better prepare future physicians to provide quality care to all patients.^11^

Although student demand is most often the inciting reason for initiating medical Spanish courses at individual medical schools,^4^ the burden of ensuring linguistic competence and patient communication skills should not fall on students. Moreover, the evidence of need is a national population health issue and should not have to be redemonstrated at individual medical schools to warrant resource allocation for course development. Similarly, heritage Spanish-speaking medical school faculty and even medical students are often relied upon to develop and teach medical Spanish courses because they are assumed to have the necessary skills to do so, but this group may feel unprepared, overburdened, and unsupported in this specialized task. Resource allocation required for course implementation may include SP or other assessment costs, faculty training, and faculty percentage time for course development and implementation. Other needs that would require institutional commitment involve providing student course credit, integration into existing communication skills portions of the curriculum, and support of interdisciplinary partnerships with language instructors who can help inform the pedagogy of language education with which clinicians may be unfamiliar.^42^

Given population trends and patient outcomes data, issues of linguistic and cultural competency have been cited by experts as an urgent call to action for educational institutions nationwide.^8,55^ Some experts have cautioned that teaching medical Spanish may increase a sense of false fluency among providers and thus create patient safety concerns that may worsen health disparities.^5,9,20,21,23,40^ We consider that the risk of false fluency may be higher for providers with some basic Spanish who have never taken a medical Spanish course and have never been taught to assess their skills or limitations, a concept supported by the finding that pediatric residents overestimated their medical Spanish proficiency before formal testing.^48^ We argue that the best approach is not to discourage medical Spanish courses altogether but rather (1) to support replication of high-quality medical Spanish educational and assessment methodology in medical schools and (2) to encourage inclusion of training for physicians on recognizing self-limitations and on appropriately accessing and working with professional interpreters when their personal skills are insufficient. This type of training has been termed global linguistic competence and defined as the skills needed to effectively communicate with patients of any language preferences or needs.^11^

The practice of medicine in English is no longer sufficient to ensure health equity for the U.S. population, so medical education institutions should develop strategies to address language discordance, including medical Spanish courses and assessment. Specifically, we propose three step-by-step recommendations for stakeholders to initiate new or improve existing medical Spanish courses, summarized in Table 1: First, we recommend to seek institutional endorsement of the role of medical Spanish education within a national health disparities context. This step is critical to ensure that medical Spanish efforts are not isolated to work done by a single educator, that they are connected to other institutional efforts to improve communication skills and care for vulnerable populations, and that they are sustainable. Second, we recommend that medical Spanish educators include precourse proficiency testing to establish a starting level for students. Precourse proficiency testing can be used to determine course eligibility and to formulate individualized student goals. Finally, we recommend that educators implement a postcourse learner examination that includes a communications skills assessment. The postcourse assessment should be nuanced enough to provide individualized recommendations regarding learner skills and limitations. By following these steps, medical educators can approach medical Spanish education as a long-term sustainable institutional effort to improve physician skills that safely and directly address population health for vulnerable linguistic minority groups in an evidence-based manner.

**Table 1.** Recommended Structural Elements for Medical Spanish Program Implementation or Enhancement

Structural elements	Recommended examples for implementation
1. Institutional endorsement of the role of medical Spanish education within a national health disparities context	a. Dedicated medical Spanish course for intermediate Spanish students or above (elective)
	b. Global linguistic skills training to understand self-limitations in any language, cultural context, and work with interpreters (all students)
2. Precourse language proficiency assessment to establish a basic fluency level for students and ensure eligibility for dedicated medical Spanish course	ILR modified scale for physicians^41^
3. Postcourse communication skills assessment	SP encounter, OSCE

ILR, Interagency Language Roundtable; OSCE, objective structured clinical examination; SP, standardized patient.

### Precourse language proficiency assessment

Although the terms competence and proficiency are often used interchangeably, we first want to establish that what is meant by proficiency is often unclear and generally should be divided in two distinct categories: (1) the assessment of precourse language proficiency, and (2) the assessment of postcourse communication skills. The purpose of the former is to determine student appropriateness to enroll in a medical Spanish course, and, as such, is a lower stakes evaluation, whereas the purpose of the latter is to determine readiness for independent direct patient care in a given language. Given these important differences, the purpose of the assessment should drive the cost and complexity of the type of testing that is recommended for each phase of the learner process. Establishing a standardized prerequisite proficiency assessment for medical Spanish courses and providing transparency as to how the Spanish postcourse proficiency will be evaluated may also serve to encourage premedical undergraduates to pursue bilingualism. For example, participating in conversational Spanish courses, seeking a Spanish minor, or pursuing other opportunities to improve general Spanish skills may enhance candidates' qualifications in later caring for Spanish-speaking populations before applying to medical school or enrolling in medical Spanish courses.

Given that medical Spanish is a complex skill that requires building upon pre-existing Spanish conversational skills, most authors agree that medical Spanish courses should focus on the advanced skill development rather than on the basic general Spanish skill acquisition.^11,14,42^ Although most medical Spanish programs rely on student self-report, and some literature supports that self-assessment may be sufficiently reliable as a way to determine starting proficiency,^56^ others have questioned its utility based on data that students may overestimate their level.^48^

A validated example of a precourse proficiency assessment that may be applied to medical Spanish courses may include the Interagency Language Roundtable (ILR) modified scale for physicians, a rapid self-assessment tool that describes proficiency categories as related to conversational health care skills in categories of “poor,” “fair,” “good,” “very good,” and “excellent.” The modified ILR scale has demonstrated accuracy at the lower and higher ends of the scale equivalent to more formal but also more costly oral language testing methodology, such as existing phone-based examinations.^41^ The American Council on the Teaching of Foreign Languages proficiency guidelines provide another scale for proficiency-level assessment for student placement, but it is not specific for health care use.^57^ It is important to emphasize that the purpose of the proficiency preassessment would be to evaluate readiness for a medical Spanish course, rather than postcourse competency or certification for clinical Spanish usage. While oral examinations or patient encounter-focused examinations to test medical language proficiency may be more reliable to provide a nuanced understanding of personal abilities, particularly for speakers in the intermediate range, these tools represent a significant time, labor, and expense, and may not be necessary for precourse assessment. A recent medical Spanish expert panel's consensus report recommends a minimum self-assessed level of “fair” or above on the modified ILR scale as a student prerequisite to enroll in a medical Spanish course in medical school.^42^

Precourse assessment, such as the modified ILR scale, can be helpful not only to determine course eligibility but also to help guide personalized learner competency goals for students at different starting levels. For example, a student at the intermediate starting level may have the goal to increase vocabulary, identify individual deficits, and complete a simple patient interview, whereas advanced students may additionally work on more complex communication skills such as informed consent discussions or delivering bad news. If an objective measure is desired that does not rely on self-assessment, a brief instructor-directed written or oral examination to evaluate general Spanish proficiency may be sufficient to establish that a student meets the course prerequisite level, although to the authors' knowledge, no specific tools have been validated for this purpose.

### Postcourse communication skills assessment

Upon completion of a medical Spanish course, a more detailed understanding of a learner's skills is necessary, including ability to self-assess language limitations and to seek help when needed. In addition, some centers are seeking to certify bilingual providers to allow them to use languages other than English in patient care, and as such are interested in a standardized certification examination that can adequately assess medical communication skills in languages besides English. Although written examinations can theoretically be used to evaluate knowledge of terminology and grammar, they do not address listening comprehension, oral communication, or interpersonal skills, which are among the most critical educational objectives of medical Spanish for physicians. Therefore, a more comprehensive and time-consuming evaluation addressing face-to-face communication skills and comprehension would be best suited for postcourse testing or certification.

Language literature supports that best practice in medical Spanish assessment should focus on oral proficiency,^39^ and medical literature provides examples of SP clinical encounters as an evaluation mechanism for clinical skills in Spanish.^43–46^ Simulation-based examinations are already the primary standard formative and summative assessment tool in U.S. medical education^58^ and are utilized with validated scales such as the Communication and Interpersonal Skills (CIS) scale in graduate competency evaluations to test U.S. medical students and residents before graduation^59^ and for licensing examinations.^60^ Similarly, in medical Spanish courses, the goal of the postcourse examination would not be focused on language proficiency alone, but rather on the provider's competence in using medical Spanish skills for patient care.

Relatedly, SP encounters can be designed appropriately for specific learners depending on skill level and course goals. Since medical Spanish proficiency level can vary depending on the complexity of the subject matter being discussed during a particular encounter and the frequency of use for a given provider, SP encounters can be used to target specific competencies in a realistic but low-stakes environment and to evaluate provider performance in various clinical situations, including difficult or high-risk patient scenarios. By contrast, a one-size-fits-all written or oral examination may not sufficiently define true competency for medical Spanish usage in clinical settings or provide sufficient guidance for providers to recognize their limitations. Interdisciplinary partnerships with professionals in other areas of language such as medical interpreters or Spanish language educators can potentially work with clinician-educators on collaboratively developing more nuanced assessments.^11^

In addition, assessment of physician language skills should acknowledge that patient-centered medical communication in a non-English language may not necessarily (and not likely) be acquired in a single course, but rather should be acquired as a longitudinal process with “graduated measures of proficiency”^21^ that physicians can develop over time. There is a need for standardization of such Spanish communication assessment examinations in connection with medical Spanish educational efforts and course objectives. In parallel, medical Spanish educators to be able should be trained to appropriately teach and assess student competencies and limitations in interviewing and caring for Spanish-speaking patients.

## Conclusions

Given that the demographics of the United Sates have continued to change substantially since the most recent national survey^4^ and that the number of U.S. medical schools has increased (154 institutions in 2019 vs. 130 in 2012), a reassessment of medical Spanish programs should be conducted. In addition, the prior survey did not query whether cultural competency or other elements of global linguistic competence (e.g., use of interpreters and understanding of basic skills in cross-linguistic communication)^11^ were included in the course instruction. Given the recognized call to action to promote adopting standardized language proficiency testing for clinicians to ensure health equity across language preferences,^8^ it would be important to assess if there has been any progress.

To facilitate new medical Spanish program implementation or improvement of existing programs, individuals who champion these efforts should consider the proposed three structural elements (Table 1) in a stepwise approach. Contextualizing medical Spanish education within larger institutional efforts to reduce population health disparities and increase physician global linguistic preparedness may facilitate inclusion of educational efforts within existing curricula and provide justification for using institutional resources. Furthemore, implementation of precourse language proficiency assessment, coupled with a comprehensive postcourse communication skills assessment, will help ensure that quality and safety measures are prioritized when teaching medical students to care for vulnerable linguistic minority patients. Importantly, attention should be given to recruitment and preparation of medical school faculty to teach and assess medical Spanish skills—a unique skill set that involves language and clinical knowledge and requires training. Additional study is needed to evaluate best practices with regard to each of the elements above including effective curricular models for medical Spanish courses, integration of global linguistic skills in existing CIS education, and development and assessment methodology of medical Spanish competencies.

The time has come to take a comprehensive approach to medical Spanish programming with the goals of making it easier for medical schools to institute effective courses for their students, to assess student communication skills with patients who prefer languages besides English, to verify effectiveness of medical Spanish programming on learner competencies, and to provide reliable and effective options for physicians to become bilingual clinicians who can offer high-quality services to the linguistically diverse U.S. population.

## Abbreviations Used

AAMC: Association of American Medical Colleges
CIS: Communication and Interpersonal Skills
ILR: Interagency Language Roundtable
SP: standardized patient

## Notes

^*^ Hereafter, the term Latino is used to refer to individuals who identify as Hispanic/Latino.

## References

[1] Vela MB, Fritz C, Girotti J Medical students' experiences and perspectives on interpreting for LEP patients at two U.S. medical schools. J Racial Ethn Health Disparities. 2016;3:245–249 27271065 10.1007/s40615-015-0134-7

[2] Hardin K. An overview of medical Spanish curricula in the United States. Hispania. 2015;98:640–661

[3] Fernández A, Pérez-Stable EJ ¿Doctor, habla español? Increasing the supply and quality of language-concordant physicians for spanish-speaking patients. J Gen Int Med. 2015;30:1394–1396 10.1007/s11606-015-3436-x PMC4579210 26209178

[4] Morales R, Rodríguez L, Singh A, et al. National survey of medical Spanish curriculum in U.S. medical schools. J Gen Intern Med. 2015;30:434–439 25862190 10.1007/s11606-015-3309-3 PMC4579233

[5] Divi C, Koss RJ, Schmaltz SP, et al. Language proficiency and adverse events in U.S. hospitals: a pilot study. Int J Qual Health Care. 2007;19:60–67 17277013 10.1093/intqhc/mzl069

[6] Improving access to services for persons with limited English proficiency. Executive Order 13166. Fed Regist. 2000;65:50119–50122

[7] Office of Minority Health U.S. Department of Health and Human Services. National standards for culturally and linguistically appropriate services in health and health care: A blueprint for advancing and sustaining CLAS—Policy and practice. Published April 2013 https://www.thinkculturalhealth.hhs.gov/pdfs/EnhancedCLASStandardsBlueprint.pdf Accessed 827, 2018

[8] Ortega P. Spanish language concordance in medical care: a multifaceted challenge and call to action. Acad Med. 2018;93:1276–1280 29877912 10.1097/ACM.0000000000002307

[9] Flores G, Mendoza FS ¿Dolor aquí? ¿Fiebre?: a little knowledge requires caution. Arch Ped Adolesc Med. 2002;156:638–640 10.1001/archpedi.156.7.638 12090826

[10] Institute of Medicine. Unequal Treatment: Confronting Racial and Ethnic Disparities in Health Care. Washington, DC: The National Academies Press, 2003 Available at https://www.nap.edu/resource/10260/disparities_providers.pdf Accessed 45, 2018 25032386

[11] Ortega P, Pérez N, Robles B, et al. Strategies for teaching linguistic preparedness for physicians: medical Spanish and global linguistic competence in undergraduate medical education. Health Equity. 2019;3: 312–318 31294243 10.1089/heq.2019.0029 PMC6615346

[12] Liaison Committee on Medical Education. Functions and structure of a medical school: standards for accreditation of medical education programs leading to the MD Degree. Effective academic year: 2019–20. Published March 2018 Available at http://lcme.org/publications Accessed 827, 2018

[13] Diamond LC, Jacobs EA Let's not contribute to disparities: the best methods for teaching clinicians how to overcome language barriers to health care. J Gen Intern Med. 2010;25 Suppl 2:S189–S193 20352518 10.1007/s11606-009-1201-8 PMC2847098

[14] Reuland DS, Frasier PY, Slatt LM, et al. A longitudinal medical Spanish program at one U.S. medical school. J Gen Intern Med. 2008;23:1033–1037 18612739 10.1007/s11606-008-0598-9 PMC2517929

[15] Diamond L, Izquierdo K, Canfield D, et al. A systematic review of the impact of patient-physician non-English language concordance on quality of care and outcomes. J Gen Intern Med. 2019;34:1591–1606 31147980 10.1007/s11606-019-04847-5 PMC6667611

[16] Ortega P, Pérez N, Robles B, et al. Teaching medical Spanish to improve population health [webinar]. Available at https://www.aamc.org/initiatives/diversity/portfolios/485628/medicalspanishwebinar.html Accessed 611, 2019 10.1089/heq.2019.0028 PMC6830530 31701080

[17] U.S. Census Bureau. Profile America facts for features: Hispanic heritage month 2016. Available at https://www.census.gov/content/dam/Census/newsroom/facts-for-features/2016/cb16-ff16.pdf Accessed 214, 2019

[18] Deville C, Hwang W, Burgos R, et al. Diversity in graduate medical education in the United States by race, ethnicity, and sex, 2012. JAMA Intern Med. 2015;175:1706–1708 26301524 10.1001/jamainternmed.2015.4324

[19] Association of American Medical Colleges. Table B-4: total U.S. medical school graduates by race/ethnicity and sex, 2013–2014 through 2017–2018. Available at https://www.aamc.org/download/321536/data/factstableb4.pdf Accessed 719, 2019

[20] Diamond LC, Tuot DS, Karliner LS The use of Spanish language skills by physicians and nurses: policy implications for teaching and testing. J Gen Intern Med. 2012;27:117–123 21773850 10.1007/s11606-011-1779-5 PMC3250531

[21] Regenstein M, Andres E, Wynia MK Appropriate use of non-English-language skills in clinical care. JAMA. 2013;309:145–146 23299604 10.1001/jama.2012.116984

[22] Huang J, Jones K, Regenstein M, et al. Talking with Patients: How Hospitals use Bilingual Clinicians and Staff to Care for Patients with Language Needs (Issue brief: Survey findings). Washington, DC: Department of Health Policy, School of Public Health and Health Services, The George Washington University, 2009

[23] Jacobs EA, Diamond LC, Stevak L The importance of teaching clinicians when and how to work with interpreters. Patient Educ Counts. 2010;78:149–153 10.1016/j.pec.2009.12.001 20036480

[24] Jacobs EA, Sadowski LS, Rathouz PJ The impact of an enhanced interpreter service intervention on hospital costs and patient satisfaction. J Gen Intern Med. 2007;22 Suppl 2:306–311 17957416 10.1007/s11606-007-0357-3 PMC2078550

[25] Karliner LS, Pérez-Stable EJ, Gildengorin G The language divide: the importance of training in the use of interpreters for outpatient practice. J Gen Intern Med. 2004;19:175–183 15009797 10.1111/j.1525-1497.2004.30268.x PMC1492142

[26] Ngo-Metzger Q, Sorkin DH, Phillips RS, et al. Providing high-quality care for limited English proficient patients: the importance of language concordance and interpreter use. J Gen Intern Med. 2007;22 Suppl 2:324–330 17957419 10.1007/s11606-007-0340-z PMC2078537

[27] Allison A, Hardin K. Missed opportunities to build rapport: a pragmalinguistic analysis of interpreted medical conversations with Spanish-speaking patients. Health Commun 2019 [Epub ahead of print]; DOI: 10.1080/10410236.2019.1567446 30706737

[28] Jaramillo J, Snyder E, Dunlap JL, et al. The Hispanic Clinic for Pediatric Surgery: a model to improve parent-provider communication for Hispanic pediatric surgery patients. J Pediatr Surg. 2016;51:670–674 26474548 10.1016/j.jpedsurg.2015.08.065

[29] DuBard CA, Gizlice Z Language spoken and differences in health status, access to care, and receipt of preventive services among US Hispanics. Am J Public Health. 2008;98:2021–2028 18799780 10.2105/AJPH.2007.119008 PMC2636430

[30] Pérez-Stable EJ, Nápoles-Springer A, Miramontes JM The effects of ethnicity and language on medical outcomes of patients with hypertension or diabetes. Med Care. 1997;35:1212–1219 9413309 10.1097/00005650-199712000-00005

[31] Parker MM, Fernández A, Moffet HH, et al. Association of patient-physician language concordance and glycemic control for limited-English proficiency Latinos with type 2 diabetes. JAMA Intern Med. 2017;177:380–387 28114680 10.1001/jamainternmed.2016.8648 PMC5339062

[32] Fernández A, Quan J, Moffet H, et al. Adherence to newly prescribed diabetes medications among insured Latino and white patients with diabetes. JAMA Intern Med. 2017;177:371–379 28114642 10.1001/jamainternmed.2016.8653 PMC5814298

[33] Hsu P, Balderas-Medina Anaya Y, Anglin L, et al. California's language concordance mismatch: clear evidence for increasing physician diversity. September 2018. Available at http://latino.ucla.edu/wp-content/uploads/2018/09/UCLA-AltaMed-Language-Concordance-Brief-2018.pdf Accessed 34, 2019

[34] García ME, Bindman AB, Coffman J Language-concordant primary care physicians for a diverse population: the view from California. Health Equity. 2019;3: 343–349 31312781 10.1089/heq.2019.0035 PMC6626968

[35] Schenker Y, Wang F, Selig SJ, et al. The impact of language barriers on documentation of informed consent at a hospital with on-site interpreter services. J Gen Intern Med. 2007;22(Suppl 2):294–299 17957414 10.1007/s11606-007-0359-1 PMC2078548

[36] Ortega P. Physicians interrupting patients. J Gen Intern Med. 2019 [Epub ahead of print]; DOI: 10.1007/s11606-019-05139-8 PMC6816729 31270788

[37] Diamond LC, Schenker Y, Curry L, et al. Getting by: under-use of interpreters by resident physicians. J Gen Intern Med. 2009;24:256–262 19089503 10.1007/s11606-008-0875-7 PMC2628994

[38] Maben K, Dobbie A Current practices in medical Spanish teaching in US medical schools. Fam Med. 2005;37:613–614 16193412

[39] Hardin KJ, Hardin DM Medical Spanish programs in the United States: a critical review of published studies and a proposal of best practices. Teach Learn Med. 2013;25:306–311 24112199 10.1080/10401334.2013.827974

[40] Diamond LC, Reuland DS Describing physician language fluency: deconstructing medical Spanish. JAMA. 2009;301:426–428 19176445 10.1001/jama.2009.6

[41] Diamond L, Chung S, Ferguson W, et al. Relationship between self-assessed and tested non-English-language proficiency among primary care providers. Med Care. 2014;52:435–438 24556893 10.1097/MLR.0000000000000102 PMC3981942

[42] Ortega P, Diamond L, Alemán M, et al. Medical Spanish standardization in U.S. Medical Schools: consensus statement from a multidisciplinary expert panel. Acad Med. 2019 [Epub ahead of print]; DOI: 10.1097/ACM.0000000000002917 31365394

[43] Ortega P, Park YS, Girotti JA Evaluation of a medical Spanish elective for senior medical students: improving outcomes through OSCE assessments. Med Sci Educ. 2017;27:329–337 29910972 10.1007/s40670-017-0405-5 PMC5998671

[44] O'Rourke K, Gruener G, Quinones D, et al. Spanish bilingual medical student certification. MedEdPORTAL. 2013;9:9400

[45] Lie DA, Forest CP, Richter-Lagha R Evaluating medical Spanish proficiency: a comparison of physician assistant student self-assessment to standardized patient and expert faculty member ratings. J Physician Assist Educ. 2018;29:162–166 30086122 10.1097/JPA.0000000000000211

[46] Mueller R. Development and evaluation of an intermediate-level elective course on medical Spanish for pharmacy students. Curr Pharm Teach Learn. 2017;9:288–295 29233415 10.1016/j.cptl.2016.11.013

[47] Reuland DS, Slatt LM, Alemán MA, et al. Effect of spanish language immersion rotations on medical student Spanish fluency. Fam Med. 2012;44:110–116 22328477

[48] Lion KC, Thompson DA, Cowden JD, et al. Impact of language proficiency testing on provider use of Spanish for clinical care. Pediatrics. 2012;130:e80–e87 22689864 10.1542/peds.2011-2794

[49] Association of American Medical Colleges. Curriculum Inventory: Coverage of Medical Spanish Education Content 2016–2017. Generated December 5, 2017

[50] Mazor S, Hampers L, Chande V, et al. Teaching Spanish to pediatric emergency physicians: effects on patient satisfaction. Arch Pediatr Adolesc Med. 2002;156:693–695 12090837 10.1001/archpedi.156.7.693

[51] Prince D, Nelson M Teaching Spanish to emergency medicine residents. Acad Emerg Med. 1995;2:32–36 7606608 10.1111/j.1553-2712.1995.tb03076.x

[52] Barr WB, Valdini A, Louis JS, et al. Sí, tú puedes: an integrated Spanish language acquisition in residency utilizing personal instruction. J Grad Med Educ. 2018;10:343–344 29946397 10.4300/JGME-D-17-00919.1 PMC6008031

[53] Association of American Medical Colleges (AAMC), Diversity in Medical Education: Facts & Figures 2016 Available at http://aamcdiversityfactsandfigures2016.org Accessed 45, 2018

[54] Martínez G, Rivera-Mills S, Trujillo JA Medical Spanish for heritage learners: A prescription to improve the health of Spanish speaking communities. Building Communities and Making Connections. Newcastle upon Tyne, United Kingdom: Cambridge Scholars Publishing, 2010, pp. 2–15

[55] Ghaddar S, Ronnau J, Saladin SP, et al. Innovative approaches to promote a culturally competent, diverse health care workforce in an institution serving hispanic students. Acad Med. 2013;88:1870–1876 24128616 10.1097/ACM.0000000000000007

[56] Reuland DS, Frasier PY, Olson MD, et al. Accuracy of self-assessed Spanish fluency in medical students. Teach Learn Med. 2009;21:305–309 20183357 10.1080/10401330903228489

[57] American Council on the Teaching of Foreign Languages Proficiency 2012 Guidelines. Available at https://www.actfl.org/sites/default/files/pdfs/public/ACTFLProficiencyGuidelines2012_FINAL.pdf Accessed 430, 2018

[58] Karkowsky CE, Chazotte C Simulation: improving communication with patients. Semin Perinatol. 2013;37:157–160 23721771 10.1053/j.semperi.2013.02.006

[59] Iramaneerat C, Myford CM, Yudkowsky R, et al. Evaluating the effectiveness of rating instruments for a communication skills assessment of medical residents. Adv Health Sci Educ Theory Pract. 2009;14:575–594 18985427 10.1007/s10459-008-9142-2

[60] Federation of State Medical Boards of the United States, Inc., and the National Board of Medical Examiners. USMLE step 2 clinical skills content description and general information. Revised October 2017 Available at www.usmle.org/pdfs/step-2-cs/cs-info-manual.pdf Accessed 828, 2018

